# Comparing the effectiveness and costs of Bevacizumab to Ranibizumab in patients with Diabetic Macular Edema: a randomized clinical trial (the BRDME study)

**DOI:** 10.1186/s12886-015-0043-x

**Published:** 2015-07-07

**Authors:** A.M.E. Schauwvlieghe, G. Dijkman, J.M. Hooymans, F.D. Verbraak, C.B. Hoyng, M.G.W. Dijkgraaf, R. Van Leeuwen, J.R. Vingerling, A.C. Moll, Reinier O. Schlingemann

**Affiliations:** Department of Ophthalmology, Academic Medical Center, University of Amsterdam, Amsterdam, The Netherlands; Ocular Angiogenesis Group, Departments of Ophthalmology and Cell Biology and Histology, Academic Medical Center, University of Amsterdam, Amsterdam, The Netherlands; Department of Ophthalmology, Leiden University Medical Centre, Leiden, The Netherlands; Department of Ophthalmology, University Medical Center Groningen, Groningen, The Netherlands; Department of Ophthalmology, Radboud University Nijmegen Medical Centre, Nijmegen, The Netherlands; Clinical Research Unit, Academic Medical Center, Amsterdam, The Netherlands; Department of Ophthalmology, University Medical Center Utrecht, Utrecht, The Netherlands; Department of Ophthalmology, Erasmus Medical Center, Rotterdam, The Netherlands; Department of Epidemiology, Erasmus Medical Center, Rotterdam, The Netherlands; Department of Ophthalmology, VU University Medical Centre, Amsterdam, The Netherlands; Department of Biomedical Engineering and Physics, Academic Medical Center, University of Amsterdam, Amsterdam, The Netherlands; Netherlands Institute for Neurosciences, Amsterdam, The Netherlands; Medical Retina Unit and Ocular Angiogenesis Group, Department Of Ophthalmology, Academic Medical Center, Room A2-122, PO Box 22660, 1100 DD Amsterdam, The Netherlands

**Keywords:** Diabetic retinopathy, Diabetic macular edema, VEGF, Ranibizumab, Lucentis, Bevacizumab, Avastin, Randomized clinical trial

## Abstract

**Background:**

The effectiveness of ranibizumab in the treatment of diabetic macular edema has been proven with large clinical trials. For bevacizumab only two clinical trials have been published and a head-to-head comparison is lacking to date. However, if proved non-inferior to ranibizumab, use of the off-label bevacizumab could reduce costs enormously without a loss in visual acuity. A cost-effectiveness study has been designed to substantiate this hypothesis.

**Aim:**

To compare the effectiveness and costs of 1.25 mg of bevacizumab to 0.5 mg ranibizumab given as monthly intravitreal injections during 6 months in patients with diabetic macular edema. It is hypothesized that bevacizumab is non-inferior to ranibizumab regarding its effectiveness.

**Design:**

This is a randomized, controlled, double masked, clinical trial in 246 patients in seven academic trial centres in The Netherlands.

**Outcomes:**

The primary outcome measure is the change in best-corrected visual acuity (BCVA) in the study eye from baseline to month 6. Secondary outcomes are the proportions of patients with a gain or loss of 15 letters or more or a BCVA of 20/40 or more at 6 months, the change in leakage on fluorescein angiography and the change in foveal thickness by optical coherence tomography at 6 months, the number of adverse events in 6 months, and the costs per quality adjusted life-year of the two treatments.

## Background

Diabetic retinopathy (DR) is the most important cause of blindness in the working age population in industrial countries. In patients with DR, diabetic macular edema (DME) is the main cause of permanent decrease of vision [1]. Until recently, the treatment options were focal and grid laser photocoagulation and intra-vitreal injections with corticosteroids [2, 3], but their efficacy is limited. The recent introduction of the anti-VEGF agent ranibizumab (Lucentis) represents an important improvement in the treatment of DME. Ranibizumab is a Fab fragment of a humanized monoclonal antibody against vascular endothelial growth factor-A (VEGF), a major causal factor in DME [4].

In several large randomized clinical trials [5–8], patients treated with ranibizumab had a better visual outcome than those treated with sham injections and/or laser therapy. Ranibizumab given as monthly injections, or in an ‘as needed’ scheme, led to a 6–10 letters better mean visual acuity after 12 months compared to control groups. The maximum effect of ranibizumab was observed around 6 months, after which the effect stabilized. In addition to its effect on visual acuity, ranibizumab markedly decreased retinal thickness as measured by optical coherence tomography (OCT) and significantly improved patient reported quality of life parameters.

Bevacizumab (Avastin) is the full length anti-VEGF-A antibody from which ranibizumab is derived [9]. Bevacizumab has been used off-label on a widespread scale by ophthalmologists in the US and Europe, and has gradually become standard care in the treatment of DME in The Netherlands, since the first results of the ranibizumab RCT’s became available in 2009. The efficacy and safety of bevacizumab 1.25 mg in the treatment of DME have been demonstrated in a number of case series and two RCTs [10]. Bevacizumab was found to improve visual acuity by approximately 8 letters at 3–12 months follow up [11, 12]. Bevacizumab markedly reduced retinal thickness on OCT, to a similar extent as reported for ranibizumab [11, 12].

Conclusive evidence from randomized controlled trials (RCT) directly comparing bevacizumab and ranibizumab is lacking.

Nepomuceno et al. recently completed a head to head comparison in 63 eyes. Patients were treated monthly if the central retinal field thickness was more than 275 μm with either bevacizumab or ranizumab for one year. They observed a significant improvement in both groups at all study visits. The improvement was significantly greater in the ranibizumab group compared with the bevacizumab group at week 8 and 32. There was no significant difference in decrease in central retinal thickness. The mean number of injections was significantly higher in the bevacizumab group (9.84) than in the ranibizumab group (7.67) [13].

At this moment two other trials are ongoing. NCT01627249 is a single blind study comparing the effectiveness of intravitreal aflibercept, bevacizumab and ranibizumaf for DME. Six hundred sixty patients will be treated over a one year time frame. The primary outcome is change in BCVA. The secondary outcome is the number of injections.

The other trial, NCT01610557 is a double blind comparison of ranibizumab as monotherapy and ranibizumab and bevacizumab consecutively.

Finally there was a double blind trial, NCT00545870, comparing bevacizumab to ranibizumab for diabetic retinopathy in 60 patients. However it has been suspended.

The costs of bevacizumab are 20 - to 40 fold lower than the costs of ranibizumab, and it has been estimated that in the Netherlands alone, the costs of ranibizumab treatment of DME would be around 10–15 million Euros higher than treatment with bevacizumab.

## Methods

### Design

The BRDME study is a multicenter, randomized, double-masked comparative clinical trial.

### Patient population

All patients with vision loss due to DME and foveal thickening (as determined on OCT) that may benefit from anti-VEGF treatment are potentially eligible for the study (Tables 1 and 2).

**Table 1:** Inclusion criteria

1. patients > 18 years of age who have signed an informed consent.
2. Type 1 or Type 2 diabetes mellitus with glycosylated haemoglobin (HbA1c) less than 12.0 % at screening. Treatment for diabetes must have been stable for at least 2 months.
3. Patients with visual impairment due to DME with a central area thickness >325 μm, who are eligible for anti-VEGF treatment according to the investigator. If both eyes are eligible, the one with the worse visual acuity, as assessed at visit 1, is selected by the investigator as the study eyes
4. BCVA equal or more than 24 and less or equal to 78 letters in the study eye at screening using ETDRS-like visually acuity testing charts at a testing distance of 4 m.

**Table 2:** Exclusion criteria

Women of child-bearing potential, unless they are using two birth control methods.
Pregnant or nursing (lactating) women.
Inability to comply with study procedures.
Active intraocular inflammation (grade + or above) in either eye at enrolment.
Any active infection in either eye at the time of enrolment.
History of uveitis in either eye at any time.
Structural damage within 600 μm of the centre of the macula in the study eye likely to preclude improvement in visual acuity following in the resolution of macular edema, including atrophy of the retinal pigment epithelium, subretinal fibrosis, laser scar(s), epiretinal membrane involving fovea or organized hard exudate plaques.
Uncontrolled glaucoma in the study eye at screening (IOP > 24 mmHg on medication or according to investigator’s judgment).
Neovascularization of the iris in the study eye.
Evidence of vitreomacular traction in the study eye.
Active untreated proliferative diabetic retinopathy in the study eye.
Any intraocular surgery in the study eye within 3 months prior to randomization.
History of vitrectomy in study eye regardless of time prior to randomization.
Planned medical or surgical intervention during the 6 months study period.
Panretinal laser photocoagulation in the study eye within 3 months prior to or during the study.
Focal/grid laser photocoagulation in the study eye 3 months prior to study entry.
Treatment with anti-angiogenic drugs in the study eye within 3 months prior to randomization.
Use of other investigational drugs at the time of enrolment, or within 3 month or 5 half-lives from enrolment, whichever is longer.
History of intravitreal corticosteroids in phakic eye within 18 months prior to randomization or in post-cataract surgery study eye within 4 months prior to randomization.
Ocular conditions in the study eye that require chronic concomitant therapy with topical ocular or systemically administered corticosteroids.
History of stroke or transient ischemic attack (TIA) within 6 months prior to enrolment.
Renal failure requiring dialysis or renal transplant or renal insufficiency with creatinine levels > 2.0 mg/dl at screening.
Blood pressure systolic > 165 mm Hg or diastolic > 105 mmHg at screening and randomization.
Hypertension or change in antihypertensive treatment within 1 month preceding randomization.
Current use of or likely need for systemic medications known to be toxic to the lens, retina or optic nerve, including deferoxamine, chloroquine/hydroxychloroquine (Plaquenil), tamoxifen, phenothiazines and ethambutol.
Known hypersensitivity to fluorescein, ranibizumab or bevacizumab or any component thereof or drugs of similar chemical classes.
Any type of advanced, severe or unstable disease or its treatment, that may interfere with primary and/or secondary variable evaluations including any medical condition that could be expected to progress, recur, or change to such an extent that it may bias the assessment of the clinical status of the patient to a significant degree or put the patient at special risk.
Concomitant conditions in the study eye which would, in the opinion of the investigator, prevent the improvement of visual acuity on study treatment.
Ocular disorders in the study eye that may confound interpretation of study results, compromise visual acuity or require medical or surgical intervention during the 6-month study period, including cataract, retinal vascular occlusion, retinal detachment, macular hole, or choroidal neovascularization of any cause (e.g., AMD, ocular histoplasmosis, or pathologic myopia)

### Randomization, blinding and treatment allocation

Included patients are randomized to receive either bevacizumab or ranibizumab.

Patient, treating physician, and evaluating investigator staff are blinded for treatment allocation. Study medication is repackaged in masked syringes at the local pharmacy.

The randomization procedure is computer- and web based, using permuted blocks and stratified by centre, BCVA of the study eye (52 letters or less versus 53 letters or more), and BCVA of the non-study eye (52 letters or less versus 53 letters or more). Randomization is made available by the AMC Clinical Research Unit. Randomization code breaking information and sheets for emergency use only is kept in the local hospital safe. Indications to break the randomization code are not predefined.

### Intervention

#### Study procedures

At the baseline visit, the patient signs the written informed consent form, and the medical and ophthalmic history is taken. Within 14 days after randomization the patient receives the first intravitreal injection of the study drug. Investigations and measurements of the BRDME trial are carried out according to the following diagram.

During each visit, vital signs (pulse and blood pressure), concomitant medication and adverse events are recorded. BCVA is assessed and an OCT examination is performed by certified personnel prior to the intravitreal injection. The interval between visits is 30 days, ±7 days to allow for flexibility in scheduling. At baseline and 6 months, an ophthalmic exam and fluorescein angiography are performed. At baseline, 3 and 6 months patients are asked to complete a short, 16-item questionnaire on health status (EQ-5D), health care resource utilization, and out-of-pocket expenses (shortened Health and Labour questionnaire) (Table 3).

**Table 3:** Study flow chart

Assessment/	Phase	Treatment							
procedure	Visit	1	2	3	4	5	6	7	8
	Month	0		1	2	3	4	5	6
Check inclusion/exclusion criteria		X							
Informed consent		X							
Medical History		X							
Vital Signs		X		X	X	X	X	X	X
Check concomitant medications		X	X	X	X	X	X	X	X
Check adverse events			X	X	X	X	X	X	X
EQ-5D or HUI-3 questionnaire			X			X			X
Blood sample		X							
Drug administration			X	X	X	X	X	X	
BCVA		X	X	X	X	X	X	X	X
Ophthalmic exam		X	X						X
Fluorescein angiography		X							X
Optical coherence tomography		X	X	X	X	X	X	X	X

### Primary outcome

The primary outcome is the mean change in best-corrected visual acuity (BCVA) in the study eye from baseline to month 6.

### Secondary outcomes

- the difference between ranibizumab and bevacizumab in their effect on retinal vascular leakage and edema as determined by FA and OCT at 6 months
- the proportions of dropouts in the two treatment arms before the final 6 months assessment
- the proportions of BCVA gainers, BCVA losers and OCT/FA non-responders in the two treatment arms at the 6 month assessment
- the difference in the occurrence of (serious) adverse events in the 6 months study period
- the difference in costs and costs per quality adjusted life-year between the two treatment strategies over the 6 months treatment period

### Ethics and funding

The study adheres to the tenets of the Declaration of Helsinki. The trial protocol has been approved by the Medical Ethical Committee of the Academic Medical Center (Amsterdam). The participation of the other centers is reviewed at each center according to Dutch law. Registration of the trial was requested June 28, 2012 at clinicaltrials.gov. NCT01635790. The study is ongoing. At the time of submission there were no publications of this trial.

The sponsor of this trial is the Netherlands Organisation for Health Research and Development, ZonMW. The study design has been peer reviewed. There is no contribution of commercial organisations.

### Data safety and monitoring board

A Data Safety Monitoring Board is installed and can recommend the steering committee of the BRDME trial to terminate the study before completion depending on the level of discrepancy between both study arms in incidences of (serious) adverse events and numbers of patients who terminate the initially assigned drug prematurely.

The DSMB members are independent and have no competing interests related to any intervention in the BRDME trial. The DSMB is composed of two members with relevant clinical expertise and one member with a background in statistics.

### Sample size

The difference in the BCVA change scores from baseline at month 6 is tested statistically for non-inferiority. Starting from a common standard deviation - based on observations in previous trials with ranibizumab in DME - of the change in BCVA score of 11 letters in both groups, and assuming an improvement from baseline of 6–8 letters in both the ranibizumab group and the bevacizumab group, a sample size of 246 patients (123 in each group) has an 80 % power of demonstrating non-inferiority by excluding a difference of 3.5 letters or more, using a one-sided Student’s *T*-test and a significance level of 0.05. The mean improvement of 7 letters is the average of the changes observed in the placebo-controlled trials discussed earlier. The margin of non-inferiority is equivalent to less than half this improvement.

### Statistical analyses

According to the intention-to-treat principle all randomized patients are included in the final analyses.

The primary outcome measure is the change in best-corrected visual acuity (BCVA) from baseline to month 6 as assessed with ETDRS-like VA charts. When visual acuity is measured in this manner, a 15 letter gain means a doubling of the visual acuity, and a 15 letter loss means that acuity is halved. As the measure of effectiveness, the mean BCVA score at month 6 in the ranibizumab group is compared with the mean score in the bevacizumab group, corrected for baseline differences. The confidence interval for the difference in mean BCVA change scores are reported. We also test statistically for non-inferiority of bevacizumab to ranibizumab using the *T*-test for independent samples.

### Ecomomic evaluation

If the non-inferiority of bevacizumab to ranibizumab can be demonstrated, then the economic evaluation will be performed as a cost-minimization analysis from a societal perspective. If bevacizumab turns out to be inferior, then the question arises whether the health losses are in reasonable balance with the expected cost savings. In that situation a cost-utility analysis will be performed with the cost per quality-adjusted life-year as outcome parameter. The analysis will be based on (i) the observed cost and visual acuity data, and (ii) available and upcoming literature on health utility associated with different levels of visual acuity. [14, 15] If a cost-utility analysis seems opportune, sensitivity analyses will be done to study the robustness of using patient-based preferences instead of general population based preferences in order to derive health utilities. The latter ones will be reported as the main outcome.

Costs will include the direct medical costs of diagnosis and treatment restricted to (potential) vision loss, including the use of visual aids. Only the medical costs attributable to loss of vision or the prevention thereof will be included in this population with a high risk of co-morbid conditions. Costs will be estimated as the product-sum of the volumes of resources used and their respective unit costs. The cost items will include visits to the health care providers (e.g., ophthalmologist, optometrists, and general practitioners), medication use, and ophthalmic equipment for imaging (Digital Imaging Systems, Fundus Camera’s, Optical Coherence Tomography) and operating theatres. Patient-related costs will include the costs of health-related travel and over-the-counter medication. In this population data on loss of productivity will also be collected. The use of resources will be documented in the case record forms and by an additional questionnaire to be completed at baseline and at 3-monthly intervals by the study participants. Unit costing will be based on the national guideline on costing in health care research, [16] supplemented by mean local unit costing data from participating reference centres and practices. Unit costs will be based on relevant national guide prices (Farmacotherapeutisch kompas, G-standaard van de Z-index).

The base year for costing will be 2013. Unit costs will be price-indexed when originating from other calendar years using general yearly price-indices [7].

The time horizon of the economic evaluation will be 6 months. No discounting of costs (and effects) will be performed.

## Discussion

Implementing a first line therapy with bevacizumab for patients with diabetic macular edema could reduce costs enormously. First, we have to investigate in a well designed head-to-head comparison whether or not both medicines are equally effective, or at least, bevacizumab is not inferior to ranibizumab. For age-related macular degeneration clinical trials have demonstrated non-inferiority [17–20] We cannot extrapolate these results to DME because the mechanism of vision loss in DME by intraretinal edema in diabetes is not the same as that of subretinal neovascularization and scarring as in macular degeneration.

We could have included aflibercept in our comparison too. At the time we designed the study there was very limited data about this medicine. We would need to enrol many more patients to be able to perform the comparison which is at present not feasible.

## Summary

In order to determine the best first line anti-VEGF medicine in the treatment of DME we decided to set up a head-to-head comparison with monthly injections during six months. The primary clinical target is the improvement of best corrected visual acuity, while the primary health economic target is cost reduction without a loss in health gain as measured by a change in visual acuity or by quality adjusted life years.

## Abbreviations

DR: Diabetic retinopathy
DME: Diabetic macular edema
RCT: Randomized clinical trial
VEGF: Vascular endothelial growth factor
OCT: Optical tomography
FA: Fluoroangiography
BCVA: Best corrected visual acuity
IOP: Intraocular pressure
TIA: Transient ischemic attack
